# Effects of Photo and Genotype-Based Misidentification Error on Estimates of Survival, Detection and State Transition using Multistate Survival Models

**DOI:** 10.1371/journal.pone.0145640

**Published:** 2016-01-11

**Authors:** Kristopher J. Winiarski, Kevin McGarigal

**Affiliations:** 1 Department of Environmental Conservation, University of Massachusetts, Amherst, Massachusetts, United States of America; 2 Northeast Climate Science Center, University of Massachusetts, Amherst, Massachusetts, United States of America; Australian National University, AUSTRALIA

## Abstract

We simulated multistate capture histories (CHs) by varying state survival (ϕ), detection (*p*) and transition (ψ), number of total capture occasions and releases per capture occasion and then modified these scenarios to mimic false rejection error (FRE), a common misidentification error, resulting from the failure to match samples of the same individual. We then fit a multistate model and estimated accuracy, bias and precision of state-specific ϕ, *p* and ψ to better understand the effects of FRE on different simulation scenarios. As expected, ϕ, and *p*, decreased in accuracy with FRE, with lower accuracy when CHs were simulated under a shorter-term study and a lower number of releases per capture occasion (lower sample size). Accuracy of ψ estimates were robust to FRE except in those CH scenarios simulated using low sample size. The effect of FRE on bias was not consistent among parameters and differed by CH scenario. As expected, ϕ was negatively biased with increased FRE (except for the low ϕ low *p* CH scenario simulated with a low sample size), but we found that the magnitude of bias differed by scenario (high *p* CH scenarios were more negatively biased). State transition was relatively unbiased, except for the low *p* CH scenarios simulated with a low sample size, which were positively biased with FRE, and high *p* CH scenarios simulated with a low sample size. The effect of FRE on precision was not consistent among parameters and differed by scenario and sample size. Precision of ϕ decreased with FRE and was lowest with the low ϕ low *p* CH scenarios. Precision of *p* estimates also decreased with FRE under all scenarios, except the low ϕ high *p* CH scenarios. However, precision of ψ increased with FRE, except for those CH scenarios simulated with a low sample size. Our results demonstrate how FRE leads to loss of accuracy in parameter estimates in a multistate model with the exception of ψ when estimated using an adequate sample size.

## Introduction

Knowledge of wildlife population dynamics is a crucial step towards species conservation and necessary if we wish to improve our understanding of the potential effects of climate change and future land use change. Accurate and precise parameter estimates of population vital rates (e.g., adult and juvenile survival rates, fecundity, etc.) are necessary for deciphering population dynamics and in forecasting future population projections. Survival (ϕ) can be estimated using capture-mark-recapture (CMR) models, but accuracy of the estimates relies on meeting model assumptions, which include: 1) individuals in a population all have an equal probability of being marked and recaptured; and 2) marks are permanent and they are observed, identified and recorded accurately at recaptures [1–3]. Traditional CMR techniques depend on physical capture, tagging and subsequent recapture, resighting or recovery. Photo and genotype-based CMR are less invasive, not requiring physical capture, but, photo-based CMR depends on focal species having unique visual markings [4–5] and genotype-based CMR relies on the presence of highly polymorphic molecular markers [6–8].

Photo and genotype-based CMR are now more feasible due to readily available software packages, which match large libraries of photo or genetic samples [4, 9–13]. With photo-based CMR software, the matching process is typically not completely automated (although see [14–15]), but relies on the user manually reviewing each photo with the most similar photos in the dataset to identify matches, with the specific measure of similarity being the major differences among software [4, 12–13]. Once photos are reviewed, individual capture histories (CHs) can be inferred from photo matches, which is required data input for CMR models. Unlike photo-based CMR, genotype-based CMR is not limited to species with distinct individual markings, as DNA samples can be collected without physical capture from hair samples or feces and with physical capture from saliva or tissues [6, 8, 16–17]. Individual identification is possible with polymorphic molecular markers, such as microsatellite loci or single nucleotide polymorphisms [8,18].

Advances with these techniques (e.g., digital photo quality for photo-based CMR and laboratory protocols for genotype-based CMR) have led to improvements in photo- and genotype- based CMR, but like the more traditional CMR approaches, they are not without error [19]. One particularly important source of error in these non-invasive CMR methods is the misidentification of true matches; i.e., failure to match a new photo or genetic sample with an existing photo or genetic sample of the same individual, leading to incorrectly concluding that it is a different individual. These "false rejections" are typically due to poor photo quality or image processing issues (e.g., significant photo glare) in the case of photo-based identification, or DNA degradation leading to false alleles or allelic dropout error in the case of genotype-based identification [8, 20]. False rejection error (FRE) is measured by estimating the percentage of known match photos or genetic samples (from the same individual) that are not identified as matches by the respective software. For photo-based CMR, FRE is a consequence of low similarity scores between matching and non-matching pairs of photos, and can differ depending on the number of top ranking photos reviewed per photo and the overall photo library size [4, 5]. For genotype-based CMR, FRE is driven by the proper selection and number of loci, PCR errors due to poor DNA quality and allele-shifting artifacts [20].

False rejection error can be as high as 25% for photo-based CMR, but is typically significantly lower for genotype-based CMR datasets [20]. Unfortunately, FRE is not usually integrated into open CMR models under the assumption that if FRE is relatively low it will not significantly bias CMR model parameter estimates, even with a number of available statistical approaches recently developed [7, 21– 24]. This is in spite of simulation findings that even low FRE will bias estimates of ϕ [5, 25]. Negative bias occurs because false rejections cause erroneous CHs resulting in a capture history with a non-detection estimate instead of a detection estimate and the creation of an additional ‘ghost history’ comprised of a single detection. Capture histories of both types contribute to lower estimates of ϕ and detection (*p*). False rejection error has previously been found to bias estimates of ϕ and *p* using simulated data, but has not been evaluated within a multistate modeling framework. Multistate models allow for estimation of an additional state transition parameter, which estimates the probability of individuals transitioning among pre-defined “states”.

Here, we simulate the effects of FRE on parameter estimation in a multistate model framework by generating multistate CHs under a gradient of realistic FRE rates. We use different scenarios of high and low ϕ and *p*, and different combinations of number of capture occasions and releases per capture occasion. Multistate models are an important class of CMR models and have been described as a unifying CMR modeling approach due to the fact that “states” can describe multiple aspects including age, geographic location, breeder or non-breeder, etc. making them applicable to a range of applications [26].

## Material and Methods

To determine the effects of photo- and genotype-based CMR FRE on accuracy, bias and precision of estimates of ϕ, p and ψ, we simulated multistate (two states) CHs under four different CH scenarios (Table 1) and numerous sample sizes (number of capture occasions and number of releases per capture occasion) using available R code [27–28] (S1 File). All CH scenarios had constant and relatively low transition probabilities between states, but the transition probability from state A to B (0.3) was set slightly higher than the transition probability from B to A (0.2) (Table 1). We simulated CHs under a scenario of 3 total capture occasions (to represent a shorter term research study) and under a scenario of 10 total capture occasions (to represent a longer term research study) along with a varying number of releases per capture occasion (25, 50, 100, 500 or 1,000) for each unique CH scenario. For each simulated CH, each individual capture had a probability of being misidentified (i.e., falsely rejected) following a Bernoulli process (0.00, 0.01, 0.05, 0.10, 0.15, 0.20 or 0.25) with estimates spanning the range of values reported in empirical studies [13]. When an error occurred, the CH was modified to reflect the error and a ‘ghost’ history was created (S1 File). For example, if we had a CH of AAAB000000, and the 2^nd^ capture was deemed to be a “false rejection” based on the Bernoulli process, then the initial CH was modified to A0AB00000 and a new ‘ghost’ capture history was also created 0A00000000. It is important to note that our simulation assumed that an individual could only be captured once and that it was not possible for a ‘ghost’ to be recaptured (S1 File).

**Table 1:** Summarized parameter values used for simulating capture histories used to evaluate effects of false rejection error on multistate model parameters.

	Multi-State Model Parameters					
Scenario	ϕ_A_	ϕ_B_	*p_A_*	*p_B_*	ψ_AB_	ψ_BA_
**high ϕ high *p***	0.90	0.90	0.90	0.90	0.30	0.20
**high ϕ low *p***	0.90	0.40	0.90	0.40	0.30	0.20
**low ϕ high *p***	0.40	0.90	0.40	0.90	0.30	0.20
**low ϕ low *p***	0.40	0.40	0.40	0.40	0.30	0.20

For each unique simulation (4 combinations of ϕ and *p* x 7 FRE rates x 5 release per capture occasion x 2 study durations = 280 unique simulations total; Table 1), we ran 1,000 iterations. Each iteration, we fit a time invariant mulitstate model (ϕ., p., ψ.) with an identity link function in program MARK [29] using the RMark package [30] for model parameterization in R [28]. Our simulation code discarded iterations where the Hessian was not positive singular or when program MARK gave a warning in respect to model convergence. We derived estimates of model parameters (ϕ_A_, ϕ_B_, p_A_, p_B_, ψ_AB_, ψ_BA_; A = state A, B = state B) using maximum likelihood [29]. We then calculated root mean square error (RMSE), a common measure of accuracy, as,

$$RMSE(\hat{\phi })=\sqrt{\frac{\sum_{i=1}^{n}{\left({\hat{\phi }}_{i}-\phi \right)}^{2}}{n-1}},$$

where $\hat{\phi }$_i_ is a survival estimate from a singe iteration, ϕ represents the true ϕ and *n* is the number of iterations. We calculated relative bias (hereafter, simply "bias") as

$$Rbi\hat{a}s(\hat{\phi })=\frac{\sum ({\hat{\phi }}_{i}-\phi )/\phi }{n}.$$

Standard error (a measure of precision) was calculated as,

$$S\hat{E}(\hat{\phi })=\sqrt{\frac{\sum_{i=1}^{n}{\left({\hat{\phi }}_{i}-\hat{\bar{\phi }}\right)}^{2}}{n-1}},$$

where $\hat{\overset{-}{\phi }}$ is the mean of the n survival estimates. We computed the RMSE, mean bias and mean standard error across the 1,000 iterations for each multistate model parameter (ϕ_A_, ϕ_B_, p_A_, p_B_, ψ_AB_, ψ_BA_) for each unique CH scenario (Table 1).

## Results

### Survival

**Fig 1:**
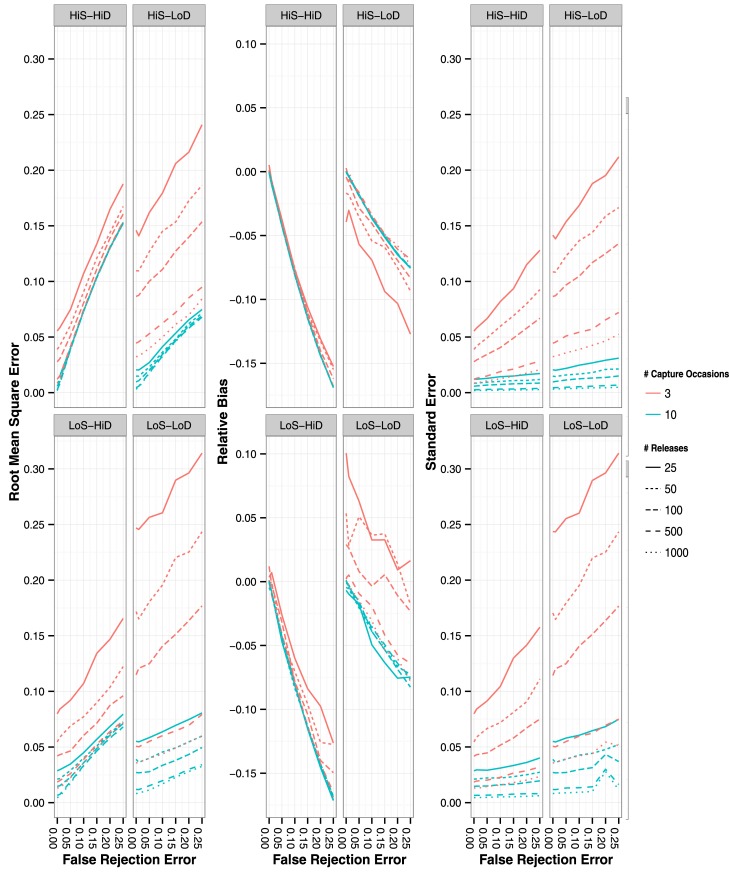
Root mean square error (left panels), residual bias (center panels) and standard error (right panels) of ϕA estimates with the four different CH simulation scenarios. False rejection rate ranged from 0% to 25%. Lines represent mean values of the 1,000 simulated iterations. Line style represents number of releases per capture occasion and line color represents number of capture occasions simulated (3 or 10 capture occasions).

As expected, ϕ decreased in accuracy with increased FRE, with lower accuracy when CHs were simulated using only 3 capture occasions and a lower number of releases per capture occasion (Fig 1; left panels). Survival of CHs simulated with 10 capture occasions decreased in accuracy with increased FRE and was lowest in those CH scenarios simulated with high *p* (Fig 1; left panels). Survival estimates were more negatively biased (with the exception of the low ϕ low *p* scenario) with increased FRE, but we found that the magnitude of bias differed by the CH scenario simulated (e.g., high *p* vs. low *p*) (Fig 1; center panels). Negative bias in ϕ was greatest with the high *p* CH scenarios (>-15% at 25% FRE) and showed a linear relationship with FRE (Fig 1; center panels). Precision of ϕ decreased with increased FRE and was lowest with the low ϕ low *p* CH scenario (Fig 1; right panels). Accuracy, bias, and precision were similar for estimates of ϕ_B_ (S1 Fig).

### Detection

**Fig 2:**
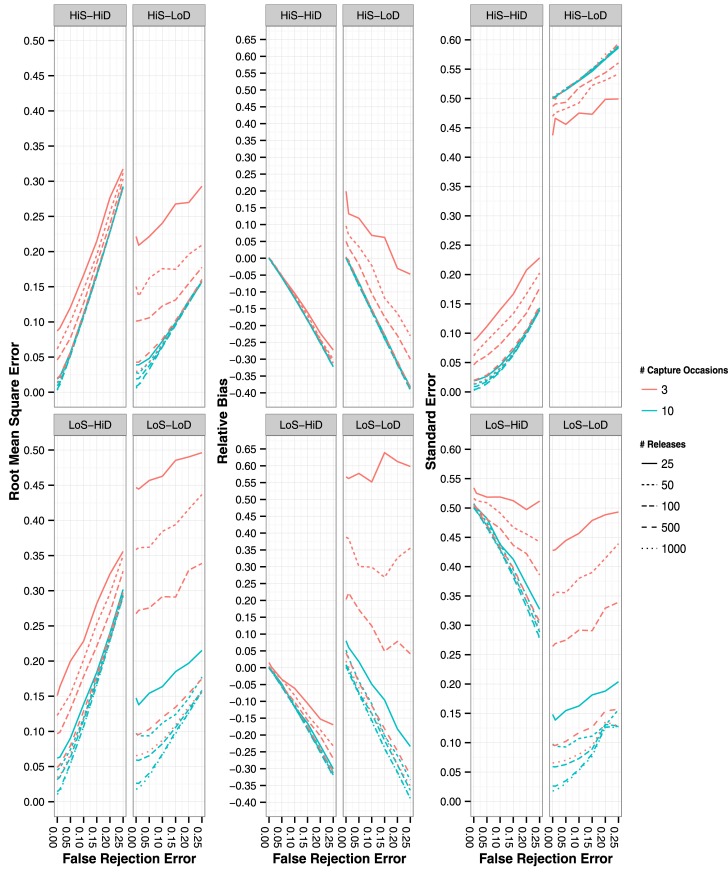
Root mean square error (left panels), residual bias (center panels) and standard error (right panels) of pA estimates with the four different CH simulation scenarios. False rejection rate ranged from 0% to 25%. Lines represent mean values of the 1,000 simulated iterations. Line style represents number of releases per capture occasion and line color represents number of capture occasions simulated (3 or 10 capture occasions).

Similar to ϕ, accuracy of *p* decreased with increased FRE and was lower with the CH scenarios simulated using only 3 capture occasions and a lower number of releases per capture occasion (Fig 2; left panels). Detection of CHs simulated with 10 capture occasions decreased in accuracy with increased FRE and was lowest in those scenarios simulated with high *p* (Fig 2; left panels). Negative bias in *p* was greatest with the low *p* CH scenarios with bias increasing with FRE (> -35% at a 25% FRE) (Fig 2; center panels). Bias in *p* was positive with the low ϕ low *p* CH scenarios simulated with low overall sample size and did not show a strong relationship with FRE (Fig 2; center panels). Detection estimates were least precise with CH scenarios with low ϕ low *p*, with precision decreasing with increased FRE under all CH scenarios, except under the CH scenario of low ϕ high *p*, where precision increased with increased FRE (Fig 2; right panels). Accuracy, bias, and precision were similar for estimates of *p*_B_ (S2 Fig).

### State-Transition

**Fig 3:**
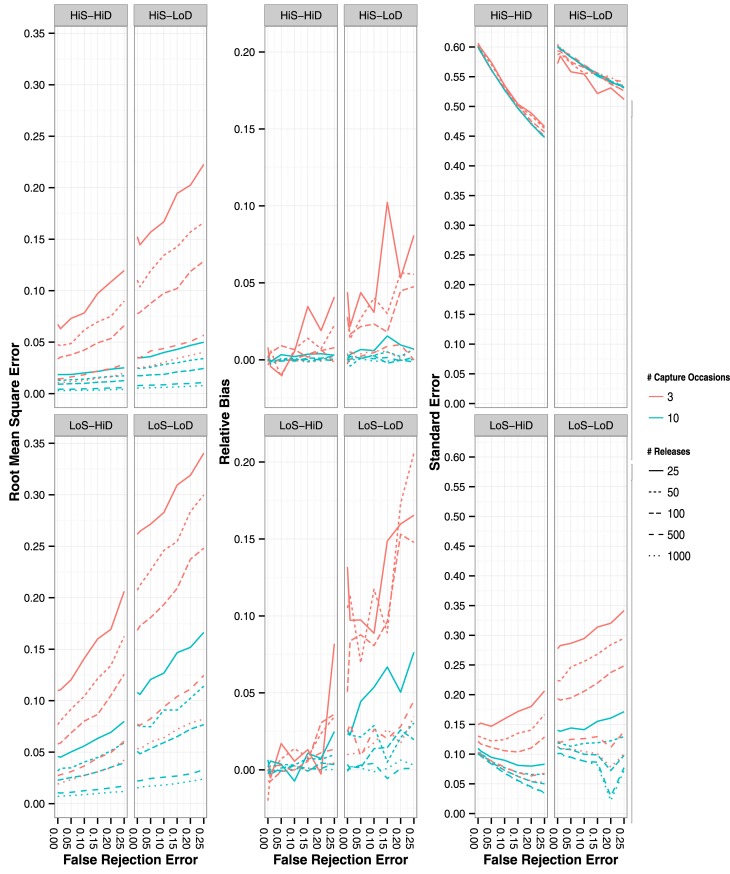
Root mean square error (left panels), residual bias (center panels) and standard error (right panels) of ψAB estimates with the four different CH simulation scenarios. False rejection rate ranged from 0% to 25%. Lines represent mean values of the 1,000 simulated iterations. Line style represents number of releases per capture occasion and line color represents number of capture occasions simulated (3 or 10 capture occasions).

Accuracy of ψ did not significantly decrease with increased FRE with CH scenarios that represented a longer-term study and had an adequate number of releases per capture occasion (Fig 3; left panels). This finding was not consistent with scenarios representing a shorter-term study simulated with a lower number of releases per capture occasion where accuracy decreased with FRE. As predicted, ψ was relatively unbiased, except with CH scenarios with low overall sample size (Fig 3; center panels). Precision of ψ was low with the high ϕ CH scenarios in comparison to the low ϕ CH scenarios and decreased with FRE (Fig 3; right panels). Accuracy, bias, and precision estimates were similar for estimates of ψ_BA_ (S3 Fig).

## Discussion

Our simulations confirmed that misidentification error, specifically, FRE, can lead to bias and reduced accuracy and precision in both state-specific ϕ and *p* (confirming results found in past simulations). False rejection error did not bias estimates of ψ (only when simulations were performed using inadequate sample sizes). Overall, the magnitude of the effect of FRE depended on the absolute value of the parameter being estimated (i.e., ϕ, *p*, or ψ), FRE rate, and the number of simulated capture occasions and number of releases per capture occasion (overall sample size). Effects of FRE and overall sample size on the accuracy, bias and precision of ϕ and *p* are of particular concern given the implications for population modeling (see below). Fortunately, precision in ϕ estimates do not appear to be overly sensitive to FRE, although is lower in those CH scenarios representing a shorter term study and a lower number of releases per capture occasions and may introduce additional uncertainty into subsequent population models (see below).

Estimates of ψ were unbiased and insensitive to FRE with the exception of CH scenarios simulated with a low number of capture occasions and number of releases per capture occasion (particularly those CH scenarios simulated with low *p*). Accuracy of ψ was also insensitive to FRE when simulated with an adequate sample size, with inaccuracy of estimates likely due to a higher number of iterations estimating ψ at the boundary and not a result of model convergence issues. To our surprise, precision of *p* and ψ estimates sometimes increased with FRE, which was counterintuitive to our initial predictions and needs to be further investigated. Our finding that ψ estimates were unbiased and relatively robust to FRE was not surprising, as ‘ghosts’ could not be recaptured in our simulation framework (transition probabilities are conditioned on individuals being captured multiple times in defined “states”). In reality, recapturing of ‘ghosts’ is likely to be extremely rare as it would be a result of false acceptance error (FAE), which is the probability of samples (e.g., photos) from different individuals being falsely matched during manual review. False acceptance error rates have previously been found to be very low and this rate will be even lower with ‘ghosts’[13].

### Reducing False Rejection Error

Our results highlight that unbiased, and more precise and accurate CMR parameters, particularly ϕ, can be achieved if FRE is relatively low (<5%) or eliminated. For photo-based CMR, FRE can be reduced by increasing processing effort per image (e.g., more precise cropping to only include relevant pattern), reducing overall photo library size and comparing results between available photo recognition software which use different techniques and algorithms for photo matching [4, 5, 12]. Although, if the difference in similarity measure between matching photos and non-matching photos is relatively high, reducing library size may not significantly decrease FRE (and may not be a practical alternative regardless) and an alternate approach may be to filter and remove low-quality photos (e.g., debris on pattern, heavy glare) to reduce overall FRE.

False rejection error with genotype-based CMR has been significantly reduced due to improvements in field protocols, laboratory procedures, and advancements with software [18, 31]. Selecting the proper and adequate number of loci is also crucial for obtaining highly confident exclusion probabilities, to ensure individuals are correctly classified. Knowledge of these loci is species-specific and better understood for some species than others. If feasible, and if a high error rate is a concern, using multiple CMR techniques (photo and genotype-based CMR) instead of just a single CMR technique may be a feasible option to reduce FRE [20].

### Incorporating False Rejection Error into CMR Models

Recently, both ad-hoc and post-hoc approaches have been developed to deal with FRE. Ad-hoc approaches include the ‘conditioning approach’, which involves filtering and discarding initial captures of non-ghosts. This approach was found to produce better estimates (in terms of RMSE) compared with ‘unconditioned’ data when FRE was >5% [5]. Unfortunately, this leads to a loss of overall data, as it requires removing CH information and results in lower precision with parameter estimates.

Post-hoc solutions to the bias caused by ghost histories seem analogous to issues caused by transients [32], although transients and residents have independent *p* probabilities, whereas ghosts and non-ghosts produced from misidentification do not [4]. Traditional CMR models for transients that assume data are drawn from multinomial distributions are inappropriate, preventing the derivation of a multinomial likelihood function [5, 33]. Recently, statistical approaches have been developed to incorporate misidentification with Bayesian, unweighted least squares and chi-square statistical approaches that perform well under certain scenarios (e.g., those where capture probabilities are high). Although potentially flexible, many existing statistical approaches incorporating FRE focus on estimating population size, rather than ϕ, with closed population models (although see [34] for an example estimating ϕ while accounting for misidentification with the robust design CMR model), but are not yet incorporated into existing CMR software packages.

### Implications for Population Modeling

Slight changes in survival rate (<5%), especially adult survival, can significantly change estimates of population growth, particularly for species with high adult survival, late maturation and few offspring [35, 36]. Bias in *p* can also have negative implications with estimating population size, which was not simulated in this study, but complements past studies looking at the effects of FRE on estimating population sizes in closed population models [7, 21, 22, 25, 34]. If ignored, bias in both ϕ and *p* can potentially lead to management decisions and actions that are based on wrong estimates. The fact that bias in ψ was relatively insensitive to FRE (except in scenarios with inadequate sample sizes) suggests that ψ may be more robust to FRE and adds to the overall broad applicability of this class of CMR models.

### Future Directions

Our simulation results are most relevant to those situations where only one sample (photo or genetic) per individual is collected per capture occasion. Multiple samples of individuals per capture occasion could theoretically lead to more accurate CHs depending on the matching protocols used. For example, allowing any new sample from the current capture occasion that matches an existing individual in the library to result in a "recapture" for that occasion (i.e., allowing multiple opportunities to confirm a match) could reduce overall FRE. Conversely, having multiple samples of the same individual that do not match individuals in the library due to poor sample characteristics could lead to higher numbers of ‘ghosts’ created per capture occasion. Exploring FRE in this context of having multiple samples from the same individual per capture occasion is important as it may lead to different levels of accuracy, bias and precision in parameter estimates and will require different statistical methods to incorporate into CMR models (although see [5, 34] for relevant examples).

Improvements also need to be made in better understanding the mechanisms behind FRE. For photo-based CMR, FRE is currently based on the percentage of known pair matches (e.g., photos matched by “eye”) that are not found to be matches by the respective matching software. In reality, the photo-recognition software outputs a relational database with photo matches (e.g., photo A and photo B do not match, but photo A and photo B match photo C, thus photo A and B match). Thus, FRE may decrease with an increased overall number of photos of the same individual or an increased number of capture occasions, where misidentification error is not necessarily due to a “bad” photo that will not rank highly with any other photos of the same individual in the dataset (as our multistate CH code simulates), but is a photo that will rank highly with other photos of the same individual and thus eventually match with a photo that it does not directly match with due to the nature of the relational database. Testing known match photos that are not constrained to just being pairwise matches (e.g., multiple photos of the same individual) could provide insight into how this error changes with overall number of photos by individual. In theory, FRE could significantly decline if there are more than a couple of photos per individual.

## Supporting Information

S1 Fig Root mean square error (left panels), residual bias (center panels) and standard error (right panels) of ϕA estimates with the four different CH simulation scenarios. False rejection rate ranged from 0% to 25%. Lines represent mean values of the 1,000 simulated iterations. Line style represents number of releases per capture occasion and line color represents number of capture occasions simulated (3 or 10 capture occasions).
(EPS)

S2 Fig Root mean square error (left panels), residual bias (center panels) and standard error (right panels) of pA estimates with the four different CH simulation scenarios. False rejection rate ranged from 0% to 25%. Lines represent mean values of the 1,000 simulated iterations. Line style represents number of releases per capture occasion and line color represents number of capture occasions simulated (3 or 10 capture occasions).
(EPS)

S3 Fig Root mean square error (left panels), residual bias (center panels) and standard error (right panels) of ψAB estimates with the four different CH simulation scenarios. False rejection rate ranged from 0% to 25%. Lines represent mean values of the 1,000 simulated iterations. Line style represents number of releases per capture occasion and line color represents number of capture occasions simulated (3 or 10 capture occasions).
(EPS)

S1 File R code for simulating capture history data, applying false rejection error and fitting multistate survival model using RMark. (PDF)
