# Supplementary material for: Effects of Photo and Genotype-Based Misidentification Error on Estimates of Survival, Detection and State Transition using Multistate Survival Models
Source: PLoS One. 2016 Jan 11;11(1):e0145640. doi: 10.1371/journal.pone.0145640 (PMC4709094; doi:10.1371/journal.pone.0145640)
Supplement: S1 File — (PDF) [file pone.0145640.s004.pdf]

```
 #R code for simulating multistage capture histories, applying a misidentification
 #error and then fitting a multistage model with RMark.

n.occasions <- 3
      n.states <- 3
      n.obs <- 3
      marked <- matrix(NA, ncol = n.states, nrow = n.occasions)
      marked[,1] <- rep(n.releases, n.occasions)
      marked[,2] <- rep(n.releases, n.occasions)
      marked[,3] <- rep(0, n.occasions)


      # Define matrices with survival, transition and recapture probabilities
      # These are 4-dimensional matrices, with
      # Dimension 1: state of departure
      # Dimension 2: state of arrival
      # Dimension 3: individual
      # Dimension 4: time

      # 1. State process matrix
      totrel <- sum(marked)*(n.occasions-1)
      PSI.STATE <- array(NA, dim=c(n.states, n.states, totrel, n.occasions-1))
      for (i in 1:totrel){
        for (t in 1:(n.occasions-1)){
          PSI.STATE[,,i,t] <- matrix(c(
            phiA*(1-psiAB), phiA*psiAB,     1-phiA,
            phiB*psiBA,     phiB*(1-psiBA), 1-phiB,
            0,              0,              1       ), nrow = n.states, byrow = TRUE)
        } #t
      } #i

      # 2.Observation process matrix
      PSI.OBS <- array(NA, dim=c(n.states, n.obs, totrel, n.occasions-1))
      for (i in 1:totrel){
        for (t in 1:(n.occasions-1)){
          PSI.OBS[,,i,t] <- matrix(c(
            pA, 0,  1-pA,
            0,  pB, 1-pB,
            0,  0,  1       ), nrow = n.states, byrow = TRUE)
        } #t
      } #i

      # Define function to simulate multistate capture-recapture data
      simul.ms <- function(PSI.STATE, PSI.OBS, marked, unobservable = NA){
        # Unobservable: number of state that is unobservable
        n.occasions <- dim(PSI.STATE)[4] + 1
        CH <- CH.TRUE <- matrix(NA, ncol = n.occasions, nrow = sum(marked))
        # Define a vector with the occasion of marking
        mark.occ <- matrix(0, ncol = dim(PSI.STATE)[1], nrow = sum(marked))
        g <- colSums(marked)
        for (s in 1:dim(PSI.STATE)[1]){
          if (g[s]==0) next  # To avoid error message if nothing to replace
          mark.occ[(cumsum(g[1:s])-g[s]+1)[s]:cumsum(g[1:s])[s],s] <-
            rep(1:n.occasions, marked[1:n.occasions,s])
```

```
  } #s
  for (i in 1:sum(marked)){
    for (s in 1:dim(PSI.STATE)[1]){
      if (mark.occ[i,s]==0) next
      first <- mark.occ[i,s]
      CH[i,first] <- s
      CH.TRUE[i,first] <- s
    } #s
    for (t in (first+1):n.occasions){
      # Multinomial trials for state transitions
      if (first==n.occasions) next
      state <- which(rmultinom(1, 1, PSI.STATE[CH.TRUE[i,t-1],,i,t-1])==1)
      CH.TRUE[i,t] <- state
      # Multinomial trials for observation process
      event <- which(rmultinom(1, 1, PSI.OBS[CH.TRUE[i,t],,i,t-1])==1)
      CH[i,t] <- event
    } #t
  } #i
  # Replace the NA and the highest state number (dead) in the file by 0
  CH[is.na(CH)] <- 0
  CH[CH==dim(PSI.STATE)[1]] <- 0
  CH[CH==unobservable] <- 0
  id <- numeric(0)
  for (i in 1:dim(CH)[1]){
    z <- min(which(CH[i,]!=0))
    ifelse(z==dim(CH)[2], id <- c(id,i), id <- c(id))
  }
  return(list(CH=CH[-id,], CH.TRUE=CH.TRUE[-id,]))
  # CH: capture histories to be used
  # CH.TRUE: capture histories with perfect observation
}

# Execute function
sim <- simul.ms(PSI.STATE, PSI.OBS, marked)
CH <- sim$CH

# Compute vector with occasion of first capture
get.first <- function(x) min(which(x!=0))
f <- apply(CH, 1, get.first)

# Recode CH matrix: note, a 0 is not allowed in WinBUGS!
# 1 = seen alive in A, 2 = seen alive in B, 3 = not seen
# Recoded CH
#rCH[rCH==0] <- 3
CH<-as.data.frame(CH)
CH$ch <- paste(CH[,1],CH[,2],CH[,3], sep = "")
ch<- CH[,4]
ch<-as.data.frame(ch)
ch[] <- lapply(ch, as.character)

# Recode CH matrix: note, a 0 is not allowed in WinBUGS!
# 1 = seen alive in A, 2 = seen alive in B, 3 = not seen
# Recoded CH
#rCH[rCH==0] <- 3
```

```
# Subset rows with >1 capture
CH <- as.data.frame(CH)
CH$V1S <- ifelse(CH$V1>=1,1,0)
CH$V2S <- ifelse(CH$V2>=1,1,0)
CH$V3S <- ifelse(CH$V3>=1,1,0)
CH$SUM <- CH$V1S + CH$V2S + CH$V3S

#Split CH into multi captures and single captures
CHsubmulti <- filter(CH, SUM>1)
CHsubmulti <- CHsubmulti[ ,-c(4,5,6,7,8)]
CHsubmulti$ID<-seq.int(nrow(CHsubmulti))

CHsubsingle <- filter(CH, SUM==1)
CHsubsingle <- CHsubsingle[ ,-c(4,5,6,7,8)]

#Remove Row ID
CHsubmulti$ID <- NULL

###Approach fragmenting a percentage of captures######
# Turn dataframe into a matrix for ease
CH_matrix <- as.matrix(CHsubmulti, rownames.force=TRUE)

# Which elements are non-zero
nonzeros <- which(CH_matrix > 0)

# What class are these events
events <- CH_matrix[nonzeros]

# Generate a vector of the same length that randomly changes some of them (say,
5%)
# into a non-event
error <- sample(0:1, size = length(nonzeros), replace=TRUE, prob = c(perror, (1-
perror)))

# Multiply the error indicator with the event type to change them, then reassign
# them into a new capture history matrix
new_events <- events * error

ghosts <- CH_matrix
ghosts[nonzeros] <- new_events

# Identify which rows have changed by calculating difference matrix
# This shows where changes were made and the class of the original event
diff <- CH_matrix - ghosts

# Finalize the ghost history

# Pull out the single changes and change row names to avoid confustion
single_change <- which(apply(diff > 0, 1, sum) == 1)
diff_single <- diff[single_change, , drop = FALSE]
if(nrow(diff_single) > 0)
  rownames(diff_single) <- paste0("ghost", 1:nrow(diff_single))

# Add single changes to the modified original
```

```
    ghosts <- rbind(ghosts, diff_single)

    # Did any capture histories experience multiple changes?  Usually a few...
    #event_changes <- arrange(data.frame(CH = rownames(CH_matrix),
    #n_changes = apply(diff > 0, 1, sum)), -n_changes)
    #head(event_changes, 10)

    # Identify these rows
    (multiple_changes <- which(apply(diff > 0, 1, sum) > 1))

    if(length(multiple_changes) > 0) {

      # Compare original to modified
      # Basically making sure I didn't screw up; looks good
      (CH_matrix[multiple_changes, ,drop=FALSE])
      (ghosts[multiple_changes, , drop=FALSE])

      # What about the capture histories with multiple event changes...
      # Here they are
      (mult_changes <- diff[multiple_changes, , drop=FALSE])

      # Find out how many ghost records we'll need for each record (usually 2, but 3
is not out of the question)
      (mults_to_add <- apply(mult_changes > 0, 1, sum))

      # Without spending a lot more time thinking about this, I'm going to loop
through these records
      # and make the necessary number of ghost histories
      # There's probably a faster way

      # Duplicate each row the correct number of times
      (diff_multiple <- mult_changes[rep(rownames(mult_changes), mults_to_add), ])

      for (CH in rownames(mult_changes)) {

        # Which records in diff_multiple are we dealing with?
        which_rows <- which(rownames(diff_multiple) == CH)

        for (row in which_rows) {
          index <- which(which_rows == row)
          elems_to_change <- which(mult_changes[CH, ] > 0)

          # Modify the record
          tmp <- mult_changes[CH, ]
          multiplier <- rep(0, length(tmp))
          multiplier[elems_to_change[index]] <- 1
          diff_multiple[row, ] <- tmp * multiplier

        }

      }

      # Change rownames (beginning where we left off) to avoid confusion
```

```
        rownames(diff_multiple) <- paste0("ghost", seq(nrow(diff_single) + 1, by = 1,
length.out = nrow(diff_multiple)))

        # Add to the ghost history record
        ghosts <- rbind(ghosts, diff_multiple)

      }

      # You can change it back to a data frame if you want
      ghosts <- data.frame(ghosts)

      ghosts$ID <- NULL

      # Recombine dataframes.
      rCH <- rbind(CHsubsingle, ghosts)  # combine the real observations with the
fragments

      # Drop empty rows
      sv <- apply(rCH, 1, sum) == 0  # if any input rows have only one obersevation
the corresponding ghost row will be empty
      rCH <- rCH[!sv, ]

      #Reformat for RMark
      rCH$ch <- paste(rCH[,1],rCH[,2],rCH[,3], sep = "")
      rCH2<- rCH[,4]
      rCH2<-as.data.frame(rCH2)
      rCH2$ch <- rCH2$rCH2
      rCH2$rCH2 <- NULL
      str(rCH2)
      rCH2[] <- lapply(rCH2, as.character)




      #Fit MS Model in RMark
      run.mstrata=function()
      {
        #
        # Process data
        #
        mstrata.processed=process.data(rCH2,model="Multistrata")
        #
        # Create default design data
        #
        mstrata.ddl=make.design.data(mstrata.processed)
        #
        #  Define range of models for S; note that the betas will differ from the
output
        #  in MARK for the ~stratum = S(s) because the design matrix is defined using
        #  treatment contrasts for factors so the intercept is stratum A and the other
        #  two estimates represent the amount that survival for B abd C differ from A.
        #  You can use force the approach used in MARK with the formula ~-1+stratum
which
        #  creates 3 separate Betas - one for A,B and C.
```

```
        #
        S.stratum=list(formula=~stratum)
        #
        #  Define range of models for p
        #
        p.stratum=list(formula=~stratum)
        #
        #  Define range of models for Psi; what is denoted as s for Psi
        #  in the Mark example for Psi is accomplished by -1+stratum:tostratum which
        #  nests tostratum within stratum.  Likewise, to get s*t as noted in MARK you
        #  want ~-1+stratum:tostratum:time with time nested in tostratum nested in
        #  stratum.
        #
        Psi.s=list(formula=~-1+stratum:tostratum)
        #
        # Create model list and run assortment of models
        #
        model.list=create.model.list("Multistrata")
        #
        # Add on specific models that are paired with fixed p's to remove confounding
        #
        #
        # Run the list of models
        #

mstrata.results=mark.wrapper(model.list,data=mstrata.processed,ddl=mstrata.ddl,thread
s=2)
        #
        # Return model table and list of models
        #
        return(mstrata.results)
      }
      mstrata.results=run.mstrata()
      #summary results for list of models run
      mstrata.results
      #model average estimates of real parameters
      model.average(mstrata.results)
      top = mstrata.results$S.stratum.p.stratum.Psi.s
      summary(top, showall = FALSE)
      head(top$results$real)
      estimates <- top$results$real$estimate[c(1,2,3,4,5,8)]
```
